# A Very Large Number of GABAergic Neurons Are Activated in the Tuberal Hypothalamus during Paradoxical (REM) Sleep Hypersomnia

**DOI:** 10.1371/journal.pone.0011766

**Published:** 2010-07-26

**Authors:** Emilie Sapin, Anne Bérod, Lucienne Léger, Paul A. Herman, Pierre-Hervé Luppi, Christelle Peyron

**Affiliations:** 1 CNRS, UMR5167, Physiopathologie des réseaux neuronaux du cycle veille-sommeil, Université Claude Bernard-Lyon 1, Université de Lyon, Lyon, France; 2 CNRS, EAC5006, Pharmacologie et Imagerie de la neurotransmission sérotoninergique, Université Claude Bernard-Lyon 1, Université de Lyon, Lyon, France; Pennsylvania State University, United States of America

## Abstract

We recently discovered, using Fos immunostaining, that the tuberal and mammillary hypothalamus contain a massive population of neurons specifically activated during paradoxical sleep (PS) hypersomnia. We further showed that some of the activated neurons of the tuberal hypothalamus express the melanin concentrating hormone (MCH) neuropeptide and that icv injection of MCH induces a strong increase in PS quantity. However, the chemical nature of the majority of the neurons activated during PS had not been characterized. To determine whether these neurons are GABAergic, we combined *in situ* hybridization of GAD_67_ mRNA with immunohistochemical detection of Fos in control, PS deprived and PS hypersomniac rats. We found that 74% of the very large population of Fos-labeled neurons located in the tuberal hypothalamus after PS hypersomnia were GAD-positive. We further demonstrated combining MCH immunohistochemistry and GAD_67_ *in situ* hybridization that 85% of the MCH neurons were also GAD-positive. Finally, based on the number of Fos-ir/GAD^+^, Fos-ir/MCH^+^, and GAD^+^/MCH^+^ double-labeled neurons counted from three sets of double-staining, we uncovered that around 80% of the large number of the Fos-ir/GAD^+^ neurons located in the tuberal hypothalamus after PS hypersomnia do not contain MCH. Based on these and previous results, we propose that the non-MCH Fos/GABAergic neuronal population could be involved in PS induction and maintenance while the Fos/MCH/GABAergic neurons could be involved in the homeostatic regulation of PS. Further investigations will be needed to corroborate this original hypothesis.

## Introduction

The brainstem is necessary and sufficient to generate paradoxical sleep (PS; also called rapid-eye-movement (REM) sleep) in cats since many aspects of PS persists after removal of the brain regions rostral to it [1], [2], [3]. PS also persists after mesencephalic trans-sections in rats [4]. However, a growing number of evidence suggests that the tuberal and mammillary hypothalamus might also contribute to PS control. Bilateral injections of muscimol in the cat mammillary and tuberal hypothalamus induce a robust inhibition of PS [5]. In addition, neurons specifically active during PS were recorded in the tuberal hypothalamus of rats [6], [7], [8], [9]. Supporting this hypothesis, we recently demonstrated using Fos immunohistochemistry as a marker of neuronal activity that the tuberal and mammillary hypothalamus contain a very large number of neurons activated during PS hypersomnia elicited by PS deprivation [10], [11]. We further found that some of these neurons were immunoreactive (-ir) to melanin concentrating hormone (MCH) and none of them were immunoreactive to hypocretin [11]. In line with these findings, it has been shown with unit recordings that the MCH and hypocretin/orexin neurons are specifically active during PS and waking, respectively [9], [12], [13]. However, the biochemical nature of the large majority of the PS-activated neurons remained to be determined.

Previous data has shown that the tuberal and mammillary hypothalamus contain a very large number of GABAergic neurons [14]. It has been suggested that the MCH neurons might co-contain GABA [15]. Therefore, to determine whether at least part of the neurons of the tuberal and mammillary hypothalamus activated during PS hypersomnia including those expressing MCH are GABAergic, we combined immunohistochemical detection of Fos with non-radioactive *in situ* hybridization (ISH) of the isoform 67 of GAD mRNA. The double-staining was performed in control rats (PSC), rats deprived of PS (PSD) with the inverted flowerpot method, and rats allowed to recover after such deprivation (PSR). We also combined immunohistochemistry of MCH with non-radioactive ISH of GAD_67_ mRNA in control rats or with Fos immunohistochemistry in PSR rats.

## Results

### Polysomnographic analysis

During the last 150 min before perfusion PSD rats displayed negligible PS amounts (0.07% (0.00 ; 0.15)) (median (Q1 ; Q3)) whereas PSC and PSR rats spent respectively 9.40% (8.17 ; 11.73) and 36.33% (36.15 ; 52.90) of their time in PS. The PSR animals spent significantly more time in PS compared to PSC and PSD rats (p<0.05). During this period, PSD animals spent significantly more time in wakefulness (64.25% (62.10 ; 68.16)) than animals from the two other groups (PSC: 40.45% (33.36 ; 48.10), p<0.05; PSR: 25.13% (17.85 ; 26.15), p<0.05). In contrast to PS and waking, the quantity of slow-wave-sleep did not differ significantly between PSD (35.72% (31.71; 38.00)) and PSR (37.10% (28.77 ; 39.10)).

### Fos distribution in the tuberal and mammillary hypothalamus

Overall, 3116 (2402 ; 4420), 1311 (1192; 1483) and 399 (193; 605) Fos-labeled neurons were counted in the tuberal and mammillary hypothalamus in PSR, PSD and PSC animals, respectively.

The tuberal hypothalamus (all structures located between −2.3 to −3.4 from Bregma, from which we excluded the ventromedial hypothalamic (VMH) and the arcuate (Arc) nuclei contained 60, 66 and 69% of the Fos-ir neurons in PSR, PSD and PSC conditions, respectively.

Among the 21 structures of the tuberal (including the Arc and VMH) and mammillary hypothalamus (all structures located between −3.4 to −5.0 from Bregma), none contained more Fos-labeled cells in PSD animals than in PSR ones. Eighteen structures contained significantly more Fos-labeled cells in PSR than PSC condition. Thirteen structures contained more Fos-labeled cells in PSD than PSC animals.

The number of Fos-labeled cells was significantly larger in PSR than in PSC and PSD conditions in 11 structures, namely the rostral zona incerta (rZI), the dorsomedial hypothalamic nucleus (DM), the medial tuberal nucleus (MTu), the tuberal part of the lateral hypothalamic area (tLH), the perifornical nucleus (PeF), the tuber cinereum (TC), the posterior hypothalamic area (PH), the VMH, the Arc, the ventral part of the premammillary nucleus (PMV) and the supramammillary nucleus (SuM). All of them, except the VMH, the Arc and the PMV, contained more Fos-ir cells in PSD condition compared with PSC.

Seven structures contained significantly more Fos-labeled cells in PSR than in PSC but not PSD conditions namely the ventral part of the zona incerta (ZIV), the subincertal nucleus (SubI), the dorsal hypothalamic area (DA), the periventricular nucleus (Pe), the mammillary part of the lateral hypothalamic area (mLH), the mammillary nucleus (M) and the submammillothalamic nucleus (SMT). These structures, except the SubI and the SMT, contained significantly more Fos-labeled cells in PSD than in PSC condition.

In the three remaining structures, the dorsal zona incerta (ZID), the dorsal part of the premammillary nucleus (PMD) and the ventral tuberomammillary nucleus (VTM), the number of Fos-ir cells did not differ across experimental conditions.

**Figure 1:**
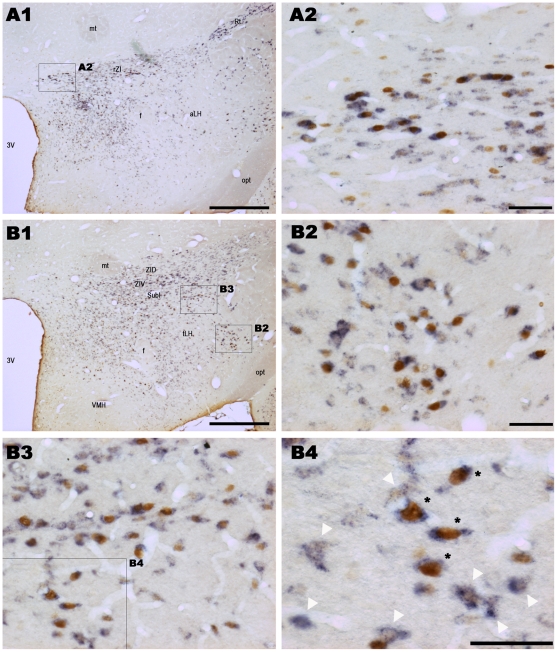
Illustration of activated GABAergic neurons in the rostral part of the zona incerta and lateral hypothalamic area after paradoxical sleep hypersomnia. Low power photomicrographs illustrating Fos-ir and GAD_67_ mRNA labeled neurons in the rostral part of the zona incerta (A1) and the lateral hypothalamic area (B1) from a PSR rat. Numerous Fos-ir (brown nuclear staining) and GAD^+^ neurons (blue diffuse cytoplasmic staining) are observed in the rZI (A2), and the LHA (B2) and (B3) at high magnification. A large part of these neurons are double-labeled. B4 is an enlargement of B3 showing Fos-ir/GAD^+^ cells (black stars) surrounded by Fos-negative/GAD^+^ cells. In agreement with previous descriptions [14], a large number of GAD_67_ mRNA labeled neurons (black) are distributed in zona incerta, the anterior and dorsomedial hypothalamic nuclei, the perifornical nucleus and the arcuate nucleus (A1, B1). A large but with a lesser density, number of GAD_67_ cells was found in the dorsal and lateral hypothalamic areas (B1). No labeling was observed in the ventromedial nucleus of the hypothalamus (B1). Abbreviations: 3V: 3^rd^ ventricle; f: fornix; aLH: lateral hypothalamic area, anterior part; mt: mammillothalamic tract; opt: optic tract; Rt: reticular nucleus of the thalamus; rZI: zona incerta, rostral part; SubI: subincertal nucleus; tLH: lateral hypothalamic area, tuberal part; VMH: ventromedial hypothalamic nucleus; ZID: zona incerta, dorsal part; ZIV: zona incerta, ventral part. Scale bars, 500µm for A1 and B1; 50µm for A2, B2 and B3 and 25µm for B4.

These results are detailed in table 1 and illustrated in figures 1–4.

**Figure 2 pone-0011766-g002:**
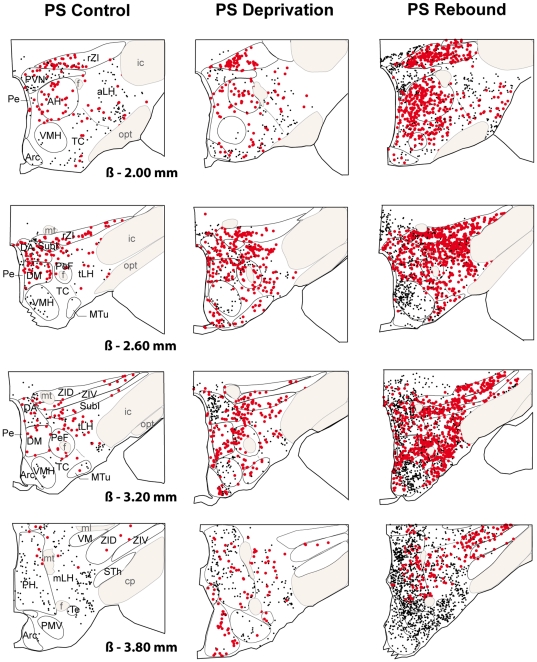
Schematic distribution of Fos-ir (small black dots) and Fos-ir/GAD^+^ (large red dots) neurons on 4 coronal sections taken at 600µm intervals in a representative animal for PSC (left hand side), PSD (middle) and PSR (right hand side) conditions after Fos immunohistochemistry combined with GAD_67_ *in situ* hybridization. Sections from −2.00 to −3.80 from Bregma (ß). Abbreviations: AH: anterior hypothalamic area; aLH: lateral hypothalamic area, anterior part; Arc: arcuate nucleus; cp: cerebral peduncle; DA: dorsal hypothalamic area; DM: dorsomedial hypothalamic nucleus; f: fornix; ic: internal capsule; ml: medial lemniscus; mLH: lateral hypothalamic area, mammillary part; mt: mammillothalamic tract; opt: optic tract; MTu: medial tuberal nucleus; Pe: periventricular nucleus; PeF: perifornical nucleus; PH: posterior hypothalamic area; PMV: premammillary nucleus, ventral part; PVN: paraventricular hypothalamic nucleus; rZI: zona incerta, rostral part; SubI: subincertal nucleus; STh: subthalamic nucleus; TC: tuber cinereum area; Te: terete hypothalamic nucleus; tLH: lateral hypothalamic area, tuberal part; VM: ventromedial thalamic nucleus; VMH: ventromedial hypothalamic nucleus; ZID: zona incerta, dorsal part; ZIV: zona incerta, ventral part.

**Figure 3 pone-0011766-g003:**
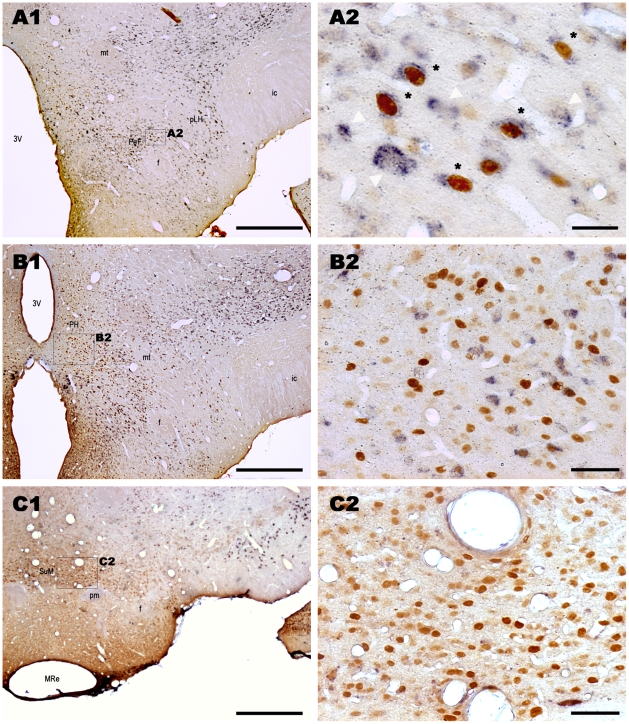
Fos-ir and GAD^+^ Single- and double-labeled neurons in the perifornical nucleus, in the posterior hypothalamic area and the supramammillary nucleus after paradoxical sleep hypersomnia. Low power photomicrographs show Fos and GAD_67_ double-labeled frontal sections at the level of the caudal part of the perifornical nucleus (A1), the mammillary hypothalamus (B1) and the mammillary nuclei (C1) in a PSR animal. Enlargement in A2 shows several Fos-ir/GAD^+^ neurons characterized by a brown nuclear and a blue diffuse cytoplasmic staining (black stars) and GABAergic singly-labeled cells (open arrows) in the perifornical nucleus. In contrast, B2 show that at mammillary hypothalamic level a majority of Fos-ir are singly-labeled in the posterior hypothalamic area. Finally, C2 shows that neurons of the supramammillary nucleus are all singly-labeled by Fos. In agreement with previous descriptions [14], only a few GAD_67_ labeled cells are located in the suprammammillary and the mammillary nuclei (C1) compared to the other tuberal and mammillary hypothalamic nuclei (A1, A2). Importantly, although the posterior hypothalamic area contains a large number of GAD_67_ labeled cells, most of them are not Fos-ir in contrast to those located more rostrally in the perifornical and lateral hypothalamic areas (A1). Abbreviations: 3V: 3^rd^ ventricle; f: fornix; ic: internal capsule; mLH: lateral hypothalamic area, mammillary part; MRe: mammillary recess of the 3^rd^ ventricle; mt: mammillothalamic tract; PeF: perifornical nucleus; PH: posterior hypothalamic area; pm: mammillary peduncle; SuM: supramammillary nucleus. Scale bars: 500µm for A1, B1 and C1; 25µm for A2 and 50µm for B2 and C2.

**Figure 4:**
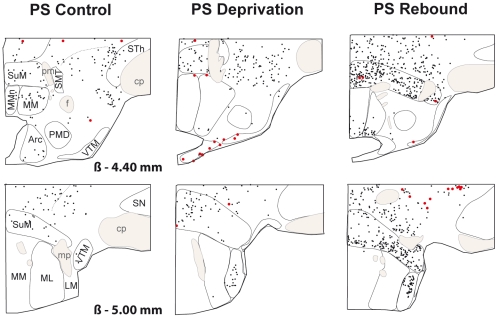
Schematic distribution of Fos-ir (small black dots) and Fos-ir/GAD^+^ (large red dots) neurons on 2 coronal sections taken at 600µm intervals in a representative animal for PSC (left hand side), PSD (middle) and PSR (right hand side) conditions after Fos immunohistochemistry combined with GAD_67_ mRNA *in situ* hybridization. Sections from −4.40 to −5.00 from Bregma (ß). Abbreviations: Arc: arcuate nucleus; cp: cerebral peduncle; f: fornix; LM: lateral mammillary nucleus; ML: medial mammillary nucleus, lateral part; MM: medial mammillary nucleus, medial part; MMn: medial mammillary nucleus, median part; mp: mammillary peduncle; pm: principal mammillary tract; PMD: premammillary nucleus, dorsal part; SMT: submammillothalamic nucleus; SN: substantia nigra; STh: subthalamic nucleus; SuM: supramammillary nucleus; VTM: ventral tuberomammillary nucleus.

**Table 1:** Number of activated neurons in Control, Paradoxical sleep deprived rats or rats with hypersomnia in paradoxical sleep.

Total Fos-ir							
	*n*	PSC		PSD		PSR	
***Tuberal Hypothalamus***							
rZI	2	55.0	(39.5 ; 71.0)	141.0*	(111.5 ; 171.8)	433.0*,#	(320.0 ; 599.0)
ZID	2	2.0	(0.8 ; 6.2)	19.5	(5.8 ; 34.0)	36.0	(11.5 ; 77.0)
ZIV	2	9.0	(6.3 ; 10.3)	23.5*	(14.3 ; 42.5)	48.0*	(25.3 ; 71.3)
SubI	2	17.0	(8.3 ; 28.0)	39.0	(35.0 ; 54.0)	70.0*	(53.5 ; 105.3)
DA	2	42.5	(19.8 ; 69.5)	166.0*	(138.5 ; 187.5)	255.0*	(198.5 ; 372.0)
DM	2	33.0	(18.3 ; 48.3)	106.0*	(97.3 ; 111.3)	183.0*,#	(156.8 ; 294.0)
Pe	3	10.5	(7.8 ; 12.3)	34.0*	(26.0 ; 44.3)	61.5*	(49.5 ; 81.8)
MTu	2	6.5	(5.8 ; 7.8)	28.0*	(27.5 ; 29.8)	127.0*,#	(96.5 ; 160.0)
tLH	2	45.5	(17.5 ; 83.8)	165.0*	(153.0 ; 173.3)	345*,#	(221.5 ; 526.3)
PeF	2	10.5	(7.8 ; 12.8)	75.0*	(62.8 ; 78.5)	162.0*,#	(157.3 ; 203.0)
TC	3	24.5	(6.8 ; 43.0)	78.0*	(63.5 ; 96.0)	192.5*,#	(167.8 ; 314.5)
VMH	3	20.0	(11.8 ; 31.8)	51.5	(43.3 ; 59.0)	240.0*,#	(173.0 ; 347.5)
Arc	4	25.0	(9.3 ; 56.3)	80.0	(71.0 ; 91.3)	184.5*,#	(164.0 ; 249.3)
***Mammillary Hypothalamus***							
mLH	1	35.0	(12.8 ; 60.8)	87.0*	(78.0 ; 107.8)	134.5*	(103.3 ; 199.5)
PH	1	43.0	(20.3 ; 67.8)	163.0*	(131.0 ; 187.3)	554.5*,#	(406.5 ; 692.0)
PMV	1	1.0	(0.8 ; 4.0)	9.0	(6.3 ; 12.5)	100.5*,#	(74.0 ; 121.3)
PMD	1	2.5	(0.8 ; 5.3)	2.5	(0.0 ; 5.3)	17.0	(11.3 ; 25.3)
M	2	6.0	(4.5 ; 11.5)	63.0*	(38.0 ; 84.3)	70.5*	(64.5 ; 75.3)
VTM	2	1.5	(1.0 ; 2.3)	7.0	(5.0 ; 9.8)	8.5	(1.5 ; 15.0)
SuM	2	18.5	(12.5 ; 28.3)	106.5*	(80.8 ; 134.5)	289.5*,#	(215.5 ; 371.5)
SMT	1	0.0	(0.0 ; 0.0)	9.5	(7.8 ; 13.5)	36.0*	(19.5 ; 54.3)

Number (median(Q1; Q3)) of Fos-ir neurons counted in the tuberal and mammillary hypothalamus in PSC, PSD and PSR rats on a total of 6 sections at 600µm intervals after Fos immunohistochemistry combined with GAD_67_ mRNA *in situ* hybridization. The values displayed are the median across 4 animals in each group of all Fos-ir neurons (Total Fos^+^) one or several sections (column *n*) depending on the rostrocaudal extent of the structures. Significance values are: *P<0.05 vs PSC; ^#^P<0.05 between PSR and PSD. Abbreviations: Arc: arcuate nucleus; DA: dorsal hypothalamic area; DM: dorsomedial hypothalamic nucleus; M: mammillary nucleus (including the lateral mammillary nucleus (LM), the lateral, medial, and median parts of the medial mammillary nucleus (ML, MM and MMn)); mLH: lateral hypothalamic area, mammillary part; MTu: medial tuberal nucleus; Pe: periventricular nucleus; PeF: perifornical nucleus; PH: posterior hypothalamic area; PMD: premammillary nucleus, dorsal part; PMV: premammillary nucleus, ventral part; rZI: zona incerta, rostral part; SMT: submammillothalamic nucleus; SubI: subincertal nucleus; SuM: supramammillary nucleus; TC: tuber cinereum area; tLH: lateral hypothalamic area, tuberal part; VMH: ventromedial hypothalamic nucleus; VTM: ventral tuberomammillary nucleus; ZID: zona incerta, dorsal part; ZIV: zona incerta, ventral part.

### Distribution of the Fos-ir/GAD^+^ double-labeled neurons in the tuberal and mammillary hypothalamus

Overall, 1591 (1298; 2274), 676 (571; 765) and 178 (104; 260) Fos-ir/GAD^+^ neurons were counted in the tuberal and mammillary hypothalamus in PSR, PSD and PSC animals, respectively (Table 2). They accounted for 44 to 52% of the total number of Fos-labeled neurons.

**Table 2:** Number of GABAergic activated neurons in Control, Paradoxical sleep deprived rats or rats with hypersomnia in paradoxical sleep.

Fos-ir/GAD+							
	*n*	PSC		PSD		PSR	
***Tuberal Hypothalamus***							
rZI	2	44.5	(30.3 ; 58.0)	128.0*	(105.5 ; 149.3)	385.0*,#	(302.0 ; 524.3)
ZID	2	2.0	(0.8 ; 4.0)	13.0	(5.0 ; 21.8)	28.0	(10.3 ; 61.5)
ZIV	2	8.0	(5.5 ; 9.5)	21.5	(12.8 ; 37.8)	46.5*	(25.3 ; 66.8)
SubI	2	11.5	(6.0 ; 20.8)	34.0	(25.8 ; 47.3)	62.5*	(50.0 ; 93.8)
DA	2	18.5	(7.8 ; 30.8)	54.5*	(46.3 ; 59.5)	106.5*,#	(91.5 ; 162.3)
DM	2	25.5	(12.8 ; 36.8)	73.0*	(68.8 ; 75.0)	116.5*,#	(103.5 ; 196.0)
Pe	3	4.5	(3.5 ; 5.5)	21.0*	(18.0 ; 23.8)	32.0*,#	(30.0 ; 42.8)
MTu	2	2.5	(1.8 ; 3.3)	16.0	(9.5 ; 20.3)	75.0*,#	(66.3 ; 90.0)
tLH	2	24.5	(11.0 ; 45.5)	99.0*	(80.3 ; 124.8)	281.0*,#	(192.3 ; 395.5)
PeF	2	9.5	(6.8 ; 11.5)	43.5*	(33.0 ; 50.5)	135.0*,#	(121.5 ; 175.0)
TC	3	7.5	(4.8 ; 14.5)	38.5	(20.5 ; 55.8)	117.5*,#	(106.5 ; 186.5)
VMH	3	0.0	(0.0 ; 0.3)	1.5*	(1.0 ; 3.5)	9.0*	(4.8 ; 17.8)
Arc	4	8.5	(2.3 ; 17.3)	40.5*	(31.5 ; 53.3)	108.5*	(75.8 ; 141.8)
***Mammillary Hypothalamus***							
mLH	1	2.0	(1.0 ; 4.5)	40.0*	(20.0 ; 70.8)	47.0*	(37.5 ; 80.8)
PH	1	4.5	(2.8 ; 10.0)	37.5*	(25.8 ; 49.8)	93.0*	(59.0 ; 147.3)
PMV	1	0.0	(0.0 ; 1.3)	2.0	(0.8 ; 3.0)	4.5	(1.5 ; 9.5)
PMD	1	0.0	(0.0 ; 0.3)	0.5	(0.0 ; 1.5)	0.5	(0.0 ; 2.0)
M	2	0.0	(0.0 ; 0.0)	1.0	(1.0 ; 1.3)	1.0	(0.8 ; 1.8)
VTM	2	0.5	(0.0 ; 1.0)	3.5	(1.8 ; 5.3)	2.5	(0.8 ; 5.8)
SuM	2	0.5	(0.0 ; 1.3)	2.5	(1.8 ; 3.3)	2.5	(1.8 ; 4.3)
SMT	1	0.0	(0.0 ; 0.0)	0.0	(0.0 ; 0.0)	0.0	(0.0 ; 0.3)

Number (median (Q1; Q3)) of Fos-ir/GAD^+^ neurons counted in the tuberal and mammillary hypothalamus in PSC, PSD and PSR rats on a total of 6 sections at 600µm intervals after Fos immunohistochemistry combined with GAD_67_ mRNA *in situ* hybridization. The values displayed are the median across 4 animals in each group of all Fos-ir/GAD^+^ double-labeled neurons counted on one or several sections (column *n*) depending on the rostrocaudal extent of the structures. Significance values are: *P<0.05 vs PSC; ^#^P<0.05 between PSR and PSD. Abbreviations are detailed in table 1.

The Fos-ir/GAD^+^ neurons constituted 74%, 66% and 62% of the total number of Fos-ir in the tuberal hypothalamus (excluding the VMH and Arc) in PSR, PSD and PSC animals, respectively (Table 3, Figs. 1, 2). In contrast, the Fos-ir/GAD^+^ represented only 16, 24 and 7% of the total number of Fos-ir in the mammillary hypothalamus in PSR, PSD and PSC rats, respectively (Table 3, Figs. 2, 3, 4). These results indicate that Fos-ir neurons located in the tuberal hypothalamus are mainly GABAergic while those located in the mammillary hypothalamus are not.

**Table 3:** Percent GABAergic activated neurons in Control, Paradoxical sleep deprived rats or rats with hypersomnia in paradoxical sleep.

		%Fos-GAD+/TotalFos-ir					
	*n*	PSC		PSD		PSR	
***Tuberal Hypothalamus***							
rZI	2	78.0	(76.1 ; 81.1)	87.5	(87.2 ; 90.0)	92.6	(89.8 ; 94.5)
ZID	2	70.6	(30.9 ; 100.0)	67.9	(50.5 ; 86.4)	81.2	(67.5 ; 91.8)
ZIV	2	95.0	(89.4 ; 100.0)	88.6	(83.6 ; 91.3)	97.7	(94.0 ; 100.0)
SubI	2	77.2	(67.7 ; 89.9)	77.9	(71.9 ; 82.6)	92.6	(91.0 ; 93.6)
DA	2	43.2	(39.4 ; 48.0)	30.5	(27.5 ; 34.9)	45.7	(41.3 ; 50.9)
DM	2	73.0	(64.7 ; 75.8)	70.7	(69.0 ; 71.0)	70.2	(66.1 ; 71.3)
Pe	3	47.2	(42.9 ; 52.1)	66.3	(51.5 ; 79.5)	61.9	(57.2 ; 64.0)
MTu	2	29.3	(26.4 ; 39.2)	51.4	(34.1 ; 62.9)	64.3	(59.8 ; 68.4)
tLH	2	57.7	(50.5 ;61.7)	68.9	(61.2 ; 81.6)	75.8	(73.2 ; 81.6)
PeF	2	89.4	(66.7 ; 90.2)	57.9	(52.5 ; 64.2)	80.2	(79.2 ; 82.7)
TC	3	48.3	(30.5 ; 66.2)	44.4	(30.8 ; 55.0)	61.1	(59.6 ; 64.0)
VMH	3	0.0	(0.0 ; 0.5)	3.9	(2.1 ; 7.4)	4.9	(2.5 ; 7.0)
Arc	4	30.4	(18.8 ; 45.7)	51.7	(40.5 ; 63.4)	55.4	(40.0 ; 62.9)
***Mammillary Hypothalamus***							
mLH	1	11.5	(6.0 ; 16.4)	44.4	(25.6 ; 50.8)	38.9	(24.9 ; 50.8)
PH	1	20.3	(11.2 ; 27.4)	24.7	(22.2 ; 26.5)	18.1	(11.0 ; 26.0)
PMV	1	0.0	(0.0 ; 9.6)	17.5	(3.8 ; 31.9)	8.4	(5.0 ; 12.2)
PMD	1	0.0	(0.0 ; 6.3)	10.0	(0.0 ; 27.5)	2.5	(0.0 ; 6.8)
M	2	0.0	(0.0 ; 0.0)	2.8	(1.2 ; 4.4)	1.6	(1.0 ; 2.6)
VTM	2	50.0	(0.0 ; 100.0)	53.3	(32.1 ; 75.0)	38.3	(20.0 ; 55.8)
SuM	2	2.4	(0.0 ; 5.6)	2.2	(1.4 ; 3.2)	0.9	(0.7 ; 1.3)
SMT	1	0.0	(0.0 ; 0.0)	0.0	(0.0 ; 0.0)	0.0	(0.0 ; 0.3)

Percent (median (Q1; Q3)) of Fos-ir/GAD^+^ neurons compare to the total number of Fos-ir cells counted on a total of 6 sections at 600µm intervals after Fos immunohistochemistry combined with GAD_67_ mRNA *in situ* hybridization. Significance values are: *P<0.05 vs PSC; ^#^P<0.05 between PSR and PSD. Abbreviations are detailed in table 1.

Eight out of the 12 structures composing the tuberal hypothalamus contained a larger number of Fos-ir/GAD^+^ in PSR than in PSD and PSC animals, namely the rZI, the DA, the DM, the Pe, the TC, the MTu, the tLH and the PeF. All these structures, except the MTu and the TC, contained a larger number of Fos-ir/GAD^+^ in PSD than in PSC animals (Table 2, Figs. 1, 2). The rZi, the PeF and the tLH contained three times more and the DA, the DM and the Pe twice as many double-labeled cells in PSR than in PSD condition (Table 2).

The last 4 structures of the tuberal hypothalamus, namely the ZIV, the SubI, the VMH and the Arc, contained significantly more Fos-ir/GAD^+^ cells in PSR than PSC animals. The difference was not significant compared to the PSD condition. In the VMH and Arc, the number of Fos-ir/GAD^+^ neurons was also significantly larger in PSD than in PSC condition.

Finally, only two of the structures of the mammillary hypothalamus had a significant number of Fos-ir/GAD^+^ neurons, namely the mLH and the PH. They both contained more double-labeled cells in PSR and PSD than in PSC animals. The number of Fos-ir/GAD^+^ did not significantly differ between PSR and PSD animals (Table 2, Figs. 2, 3, 4).

### MCH immunohistochemistry and GAD_67_ mRNA double-staining

**Figure 5:**
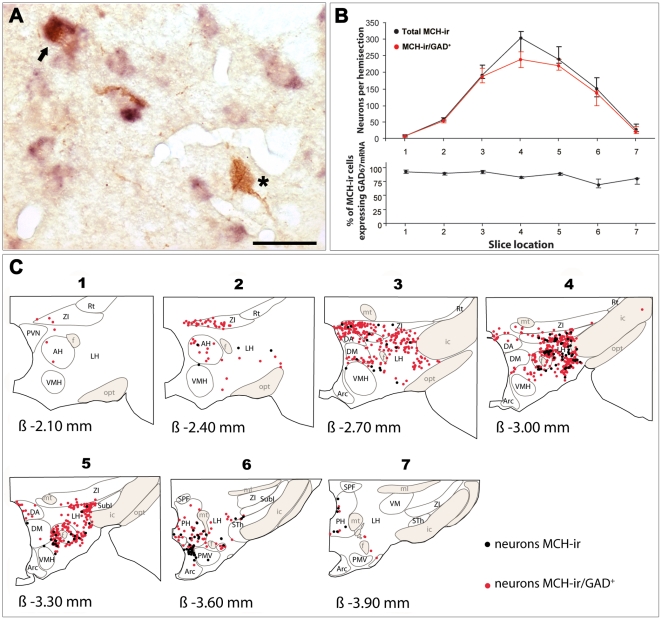
Colocalization of MCH neuropeptides and GAD_67_ mRNA in neurons of the tuberal hypothalamus. (A) Photomicrograph showing one MCH-ir cell (black star, brown cytoplasmic staining only) and one MCH-ir cell co-expressing GAD_67_ mRNA (black arrows, brown and blue diffuse cytoplasmic stainings superimposed) in the lateral hypothalamic area. (B) Graphics illustrating the distribution of MCH-ir cells along the rostrocaudal axis (from the first to the 7^th^ section). The upper graph reports the average number of MCH-ir cells (black dots) (median ± Q1;Q3) and of MCH-ir/GAD^+^ neurons (red dots) counted on hemisections from three rats. The bottom graph illustrates the percent of MCH-ir/GAD^+^ cells over the total number of MCH-ir neurons on each hemisection. (C) Schematic distribution of MCH-ir (black dots) and MCH-ir/GAD^+^ (red dots) neurons in on representative rat brain. The approximate planes of sections relative to Bregma (β), according to the atlas of Paxinos and Watson [51], are −2.10 (1), −2.40 (2), −2.70 (3), −3.00 (4), 3.30 (5), 3.60 (6) and −3.90 (7). Scale bar: 30 µm. Abbreviations: AH: anterior hypothalamic area; Arc: arcuate nucleus; DA: dorsal hypothalamic area; DM: dorsomedial hypothalamic nucleus; f: fornix; ic: internal capsule; LH: lateral hypothalamic area; ml: medial lemniscus; mt: mammillothalamic tract; PH: posterior hypothalamic area; PMV: premammillary nucleus, ventral part; PVN: paraventricular hypothalamic nucleus; opt: optic tract; Rt: reticular thalamic nucleus; SPF: subparafascicular thalamic nucleus; STh: subthalamic nucleus; SubI: subincertal nucleus; VM: ventromedial thalamic nucleus; VMH: ventromedial hypothalamic nucleus; ZI: zona incerta.

As reported by us and others [16], [17], most of the MCH-ir cells were localized in the tuberal hypothalamus. Most of them were located in the ZI, the LH, and the PeF (Fig. 5). Some cells were scattered throughout the DM and the PH. The region containing MCH cells extended over a rostro-caudal distance of 2 mm (−2.10 mm to −3.90 mm from Bregma) (Fig. 5*B,C*).

A large proportion of MCH-ir cells, 841 (829 ; 876.5) neurons out of 1005 (997 ; 1020.5); 85% (83.2 ; 86.5) was found to express GAD_67_ mRNA (Fig. 5). No segregation of double-labeled cells in a given area was observed (Fig. 5*C*). Indeed, after counting and expressing graphically the number of plotted cells per hemisection, we found that the largest numbers of MCH-ir cells were distributed in sections extending from −2.70 to −3.60 mm from Bregma (Fig. 5*B, C*) and that the MCH-ir cells co-expressing GAD_67_ mRNA closely followed the same pattern of distribution (Fig. 5*B*).

### Numbers of Fos-ir/MCH^+^, MCH^+^/GAD^+^ and Fos-ir/GAD^+^ double-labeled neurons

To determine the proportion of MCH and non-MCH Fos-ir/GAD^+^ neurons, counts of neurons were made on two hemi-sections (−2.7 and −3.3 mm from Bregma) double-stained for Fos/MCH or Fos/GAD in PSR animals and for MCH/GAD in control animals.

We found a total of 1467±420 Fos-ir/GAD^+^, 414±7 MCH^+^/GAD^+^ (47±16 MCH^+^/GAD−) and 293±30 Fos-ir/MCH^+^ (214±20 single MCH^+^) double-labeled neurons in the tuberal hypothalamus.

These results indicate that no more than 20% (293/1467) of the Fos-ir/GAD+ neurons of the tuberal hypothalamus contain MCH.

## Discussion

We report here for the first time that 74% of the very large number of Fos-labeled cells located in the tuberal hypothalamus after PS recovery express GAD_67_ mRNA. We also demonstrate that 85% of the MCH neurons express GAD_67_ mRNA. We uncovered that approximately 20% of the GABAergic neurons activated in PS recovery contain MCH. In contrast, only a small percentage of the numerous Fos-labeled neurons localized in the mammillary hypothalamus expressed GAD_67_ mRNA. After discussing methodological issues, we will compare our data with previous studies and discuss the potential role of such an important neuronal population in the control of PS.

### Technical considerations

Despite the fact that Fos immunohistochemistry was combined with GAD ISH, the distribution and number of Fos-ir labeled neurons in the tuberal and mammillary hypothalamus observed here were similar to those reported in our previous work using the same Fos antiserum and PS deprivation/recovery method [10]. The distribution of GAD-labeled neurons was also in agreement with previous studies [14], [18]. These observations confirm that our double-staining method combining Fos immunostaining with GAD_67_ ISH is well suited to reveal activated GABAergic neurons.

Fos is an immediate early gene with a fast and transient induction curve in activated neurons and a low level of expression under basal condition [19]. The half-life of the Fos protein is of approximately 40 min and less than 5% of protein remains 3 hours after stimulation [20], [21]. It is therefore a good tool to map neurons activated in response to pharmacological stimuli and physiological challenges. The main criticism of this method is that Fos expression is not strictly correlated with neuronal activity [22]. We cannot rule out that some activated neuronal populations are not seen. However, the identification of neurons specifically active during PS using Fos method was confirmed by single-unit recordings in the three structures examined so far. Indeed, neurons discharging specifically during PS were recorded in the tuberal hypothalamus [8], [23] some of them being characterized as MCH neurons [9], in the dorsal paragigantocellular nucleus [24] and in the sublaterodorsal tegmental nucleus [25]. Furthermore, Fos immunostaining has been extensively used in recent years to identify the neuronal network of PS [10], [11], [17], [26], [27], [28], [29], [30], [31], [32], [33], [34]. Another recurrent criticism of our approach is that the inverted flowerpot PS deprivation method disrupts energy balance and induces hyperphagia [35] and chronic stress [36]. However, a more recent report indicates that hyperphagia is observed after 5 days of PS deprivation and not after 72 hours [37]. We are aware that some neurons might be Fos-labeled after PS deprivation due to the experimental conditions. Complementary electrophysiological recordings using juxtacellular labeling would be needed to confirm that the Fos-ir/GAD^+^ neurons discharge specifically during PS as done previously with other structures [9], [12]. The Fos-labeling method is nevertheless very interesting since in contrast to electrophysiological recordings, it provides a full map of the activated neurons. Although the PS deprivation method and the Fos immunostaining have their drawbacks, the combination of the two is still the only available method to map PS-inhibitory and PS-executive structures.

### Functional significance

The most noteworthy result of the present study is the very large number of Fos/GAD^+^ double-labeled neurons counted in the tuberal hypothalamus in PSR animals. When comparing the results with data recently published on the brainstem [38], it is interesting to emphasize that, in PSR animals, the tuberal and mammillary hypothalamus contained 2.5 times more Fos-ir/GAD^+^ labeled neurons than the entire brainstem. Such a comparison can be carried out because the same animals were used in both studies and the brainstem and hypothalamic sections were histologically processed at the same time. This observation is surprising because pioneer studies of trans-sections in cats showed that PS persists after removal of the brain regions located rostral to the brainstem indicating that the brainstem is necessary and sufficient to generate PS [1], [2], [3]. Nevertheless, it is in agreement with the fact that the inactivation of the tuberal and mammillary hypothalamus by muscimol applications almost completely abolishes PS [5]. These and the present results suggest that although the basic machinery for generating PS is contained within the brainstem, GABAergic neurons of the tuberal hypothalamus play an important role in the regulation of PS.

We previously found that a significant number of the Fos-labeled neurons located in the tuberal hypothalamus in PSR animals are immunoreactive to MCH [11], [17]. We report here that 74% of the Fos-labeled neurons located in the same region in PSR animals express GAD_67_ mRNA. Although it was suspected, we further provide the first direct demonstration that most of the MCH neurons (85%) express GAD_67_ mRNA. Altogether, our results reveal the existence of two sub-populations of GABAergic neurons active during PS: a large population of non-MCH/GABAergic neurons (≈4/5 of the Fos-ir/GAD^+^), and a population of neurons co-containing MCH and GABA (≈1/5 of the Fos-ir/GAD^+^), both populations being intermingled in the tuberal hypothalamus.

It has recently been shown using juxtacellular recordings that the MCH neurons fire only during PS and start firing at the onset of PS [9]. We thus propose that MCH/GABAergic neurons are involved in the maintenance of PS and not in its induction [39]. Interestingly, ICV administration of MCH [11] and local injection of MCH within the dorsal raphe [40] induce a strong increase in PS quantity while subcutaneous injection of MCH receptors antagonists induces a decrease in PS quantity [41]. Altogether, these data suggest that the MCH/GABAergic neurons play a crucial role in the homeostatic regulation of PS (for review see [39]).

Regarding the large population of non-MCH/GABAergic neurons found in the present study, it is likely that they also fire specifically during PS. In support of this hypothesis, a large number of neurons with specific activation during PS and displaying a much higher firing rate during PS than the MCH neurons were recorded in the tuberal hypothalamus in head-restrained rats [8], [23]. Furthermore, in contrast to the MCH neurons, many of these PS-active neurons increased their firing rate prior to the onset of PS suggesting that they are able to maintain but also to induce PS. Although it is speculative and many more studies need to be carried out, we propose that the non-MCH/GABAergic neurons belong to this class of neurons and may be responsible for the inhibition of the local hypocretin and histaminergic neurons at the onset and during PS. In return, the GABAergic neurons that were found in the present study to be Fos-positive after PS deprivation may inhibit the local PS-active GABAergic neurons during waking and slow-wave-sleep and thus prevent the onset of PS during deprivation.

In summary, we propose that non-MCH/GABAergic neurons of the tuberal hypothalamus active during PS would play a key role in PS induction and maintenance by means of their inhibition of the co-localized wake-active hypocretin and histaminergic neurons. The MCH/GABAergic neurons may play a role in PS homeostasis via their projections to wake-active monoaminergic neurons, slow-wave-sleep-active neurons of the ventrolateral preoptic area and the PS-inhibitory GABAergic neurons of the brainstem vlPAG/dDPMe.

## Materials and Methods

Sprague–Dawley male rats were housed under a constant light/dark cycle (lights on from 7:00 AM to 7:00 PM). The room temperature was maintained at 21±1°C and standard rodent food and water were available *ad libitum* throughout the experiment.

### Study of activated neurons after PS deprivation or hypersomnia

Eighteen rats (200–230g, Charles River, France) were implanted for electroencephalogram (EEG) and electromyogram (EMG) recording and were subjected to a PS deprivation/hypersomnia protocol. Animals were then perfused with a fixative solution and brains were removed and sliced in coronal sections. Experimental protocols performed on animals were approved by the institutional animal care and use committee of the University of Lyon 1 (protocol BH 2006–09; BH 2006–10).

#### EEG and EMG recording

Animals were implanted with EEG and EMG electrodes under chloral hydrate anesthesia (400 mg/kg, i.p.). Four stainless steel screws were fixed in the occipital, parietal, and frontal parts of the skull and two wire electrodes were inserted into the neck muscles. All leads were connected to a miniature plug that was cemented to the skull. After surgery, rats were placed on a bed of woodchips in a 30 cm diameter, 40 cm height Plexiglas jar for the duration of the experiment. Animals recovered from surgery for 5 days before being transferred to the recording chamber where they became accustomed to the recording conditions (environment and cable) for the next 4 days.

For polygraphic recordings, rats were connected to a cable attached to a rotating connector to allow free movements of the animal within the jar. EEG and EMG bipolar signals were amplified, digitized at 250 Hz and collected with CED using Spike-2 interface software (Cambridge Electronic Design, Cambridge, UK). Hypnograms were scored off-line manually by using cortical EEG and nuchal EMG. The episodes of each vigilance state were scored by using a 10-sec sliding time window according to the following criteria: wake was characterized by desynchronized low-voltage and high-frequency activity EEG and by a sustained EMG neck muscle tone; slow-wave-sleep was characterized by high-voltage slow waves (1.5–4 Hz) and spindles (10–14 Hz) combined to a low muscle tone; PS episodes were identified by a decrease in the EEG amplitude and a prominent theta rhythm (5–9 Hz) associated with a muscle atonia on the EMG. For 4 PSC, 5 PSD and 7 PSR rats, the last 150 min of EEG/EMG recordings before sacrifice were analyzed in order to determine time spent in each vigilance state and the number and duration of wake, slow-wave-sleep and PS bouts.

#### Paradoxical sleep deprivation and recovery

PS deprivation was performed using the inverted flowerpot technique that has been previously shown to induce a fairly selective deprivation of PS in rats [10], [11], [28], [29], [33], [38], [42]. This technique is known to cause no significant change in adrenal gland weights [43], a measure of stress level in animals. Rats were divided into 3 groups: control (PSC; n = 5), deprived of PS (PSD; n = 5), and PS recovery (PSR; n = 8). The PSC animals remained on a bed of woodchips in the recording room throughout the experiment. After 48 hours of baseline recording, PSD and PSR rats were placed (9:00 AM) on a platform surrounded by 1–2 cm of warm water (∼33°C) for 72 h. The platform was just large enough (6.5 cm in diameter) to hold the animal. In this setting the animal could engage in slow-wave-sleep but not PS because of the loss of muscle tone that occurs during PS. The platform was well above the water (9 cm height) to limit tail contacts with water. Each morning (between 9:00 and 10:00 A.M.), in order to clean their jar, rats were removed from their platform and placed in another Plexiglas jar where they could groom and stretch. On the last day, PSR animals were removed from the platform (10:00 A.M.) and were returned to a dry bed of woodchips in their recording jars to allow PS recovery (generally occurring after ∼30 min of exploration and grooming). PS rebound can last for several hours after 72 h of PS deprivation. The total amount of PS during the first 6 hours of rebound is approximately 30% whereas it accounts for up to 40% in the first 3 hours (personal observation). Furthermore, synthesis of the Fos protein is maximal 1–2 hours post-stimulation [44]. Based on these data, we chose to let rats recover for exactly 150 min after the first episode of PS. During that time, PSD rats were kept sleep deprived on the platform.

Finally, PSC animals were anesthetized for perfusion (at ∼1:00 P.M.) followed by PSD rats, then PSR rats.

#### Perfusion, fixation and section preparation

Under profound barbiturate anesthesia (pentobarbital, Ceva santé animale, 150 mg/kg, i.p.), rats were transcardially perfused with Ringer's lactate solution containing 0.1% heparin followed by 500 ml of a fixative solution composed of 4% paraformaldehyde in 0.1 M phosphate buffer (PB, pH 7.4). Brains were stored overnight at 4°C in the fixative solution then in 30% sucrose in 0.1 M PB for 3 days. After that, they were rapidly frozen with CO_2_ gas and 30 µm-thick coronal sections were cut on a cryostat. The free-floating sections were collected and stored at −20°C in an RNase-free cryoprotective solution (0.05% DEPC, 20% glycerol, 30% ethylene glycol in 50 mM PB, pH 7.4).

### Fos immunohistochemistry combined with GAD_67_ mRNA *in situ* hybridization

The optimal labeling of the tuberal hypothalamus' GABAergic neurons by immunohistochemistry is very difficult and requires the use of colchicine to block axonal transport [18]. This technique is not compatible with sleep deprivation experiments. Therefore, GAD was detected by GAD_67_ *in situ* hybridization.

As described before [38], [45], the antisense probe was obtained after linearization of the recombinant plasmid containing the GAD_67_ cDNA [46] with EcoRV and *in vitro* transcription using SP6 RNA polymerase and a non-radioactive digoxigenin (DIG) RNA labeling kit (Roche Diagnostic, Mannheim, Germany).

Brain sections were successively incubated in a rabbit antiserum to Fos (1∶4000; Ab-5; Oncogene, CA, USA) in 10 mM PB containing 0.9% NaCl and 0.3% Triton-100X (PBST) for 18h followed by a biotinylated goat anti-rabbit IgG solution (1∶1000; Vector Laboratories, Burlingame, CA, USA) and then an ABC-HRP solution (1∶1000; Elite kit, Vector Laboratories) both for 90 min. Finally, the sections were immersed in a 0.05 M Tris-HCl buffer (pH 7.6) containing 0.025% 3.3′-diaminobenzidine-4 HCl (DAB, Sigma-Aldrich, St. Louis, MO, USA) and 0.003% H_2_O_2_. Three washes of 10 min in PBST were performed between each step. The sections were then rinsed two times in PBST containing 10 mM dithio-threitol (DTT, Sigma-Aldrich) for 10 min and then in standard saline citrate solution (SSC 2X) for 10 min. All buffers except for PBST with DTT contained 0.2% of RNase inhibitor (solution Protect RNA RNase inhibitor, Sigma-Aldrich).

After these rinses, the sections were placed for 18 h at 65°C in a hybridization buffer made of NaCl (150 mM), Tris-HCl (8 mM), Tris-Base (1 mM), NaH_2_PO_4_ (6 mM), Na_2_HPO (5 mM), EDTA (5 mM), formamide (50%), dextran sulphate (10%), yeast tRNA (Sigma type III, 1 mg/ml, Sigma-Aldrich), ficoll (0.02%), and polyvinylpyrrolidone (0.02%) containing 0.5 µg/ml of the DIG-labeled probe. After hybridization, the sections were washed in a 1X SSC, 50% formamide, 0.1% Tween-20 solution at 55°C twice for 20 min and treated with 10 µg/ml RNase A (USB, Cleveland, OH, USA) in Tris 10 mM (pH 8.0) containing 1 mM EDTA and 500 mM NaCl for 15 min at 37°C. They were then rinsed 3×10 min with PBST. For immunohistological detection of DIG, the sections were incubated overnight with anti-DIG conjugated to alkaline phosphatase (Roche Diagnostic) diluted 1∶2000 in PBST containing 0.2% blocking agent (Roche Diagnostic). After incubation, the sections were washed in PBST twice for 10 min and then in a buffer containing 100 mM Tris (pH 9.5), 100 mM NaCl and 50 mM MgCl_2_. The sections were developed at 37°C for ∼4 h in the same buffer with nitroblue tetrazolium (NBT) and 5-bromo-4-chloro-3-indolyl-phosphate (BCIP) diluted 1∶50 from the stock solution (Roche Diagnostic). Incubations were performed at room temperature unless otherwise noted. Finally, the sections were mounted on glass slides, dried and coverslipped with Vectamount (Vector Laboratories). Controls in the absence of primary antibodies (anti-Fos and anti-DIG) and with the sense probe (GAD_67_ cDNA linearised with HindIII restriction enzyme and transcribed using T7 RNA polymerase) were run to ensure the absence of nonspecific labeling. As specified by the supplier, the Fos antiserum was made against a synthetic peptide corresponding to the N-terminal part (residues 4–17) of human Fos protein. This part of the protein displays 100% homology between humans and rats [47], [48] and no homology with Fos-related antigens such as Fos B, Jun B, Fra-1 and Fra-2 (Blast 2 sequences, NCBI). In agreement with previous studies, very few immunoreactive nuclei were observed with this Fos antiserum in rat brains following a control perfusion during daylight [49], [50]. Brains from sets of PSC, PSD, and PSR animals that were run together during the deprivation/rebound part of the experiment were processed together for immunohistochemistry and ISH.

### Immunohistochemistry of Fos and MCH

We previously showed that MCH neurons are activated only after PS rebound [11], [17]. We therefore performed this double-labeling on PSR rats (n = 4) only. Sections were first processed for Fos immunohistochemistry as mentioned above except that 0.6% nickel ammonium sulphate was added to the DAB solution. The Fos stained sections were incubated the following day in a rabbit antiserum to MCH (1∶100,000; Phoenix Pharmaceutical) in PBST-Az over 3 days at 4°C. Amplification steps were similar to those used for Fos but revelation was performed in DAB solution without nickel [11], [17].

### Immunohistochemistry of MCH combined with GAD_67_ mRNA *in situ* hybridization

Three additional male Sprague-Dawley rats (270–280g, Charles River, France) underwent the same fixative protocol as described above. Brains were removed and cut in 25 µm-thick coronal sections. They were processed for MCH immunohistochemistry followed by GAD_67_ ISH using the same method as above. The only difference was the use of a rabbit antiserum against MCH (1∶5000; Phoenix Pharmaceuticals, Burlingame, CA, USA) instead of the Fos antibody.

### Analysis of immunohistochemical data

Despite the fact that we tried all possible combinations, we were not able to combine the labeling of Fos, GAD and MCH on the same sections. To tackle this problem, we used several double stains on different sections. The distribution of the Fos-ir, Fos-ir/GAD^+^, Fos-ir/MCH^+^, MCH-ir and MCH-ir/GAD^+^ labeled neurons was similar between the two hemispheres of the brain. As such, only hemi-sections were analyzed. Drawings of double-labeled sections were made with an Axioskop microscope (Zeiss, Germany) equipped with a motorized X–Y-sensitive stage and a video camera connected to a computerized image analysis system (Mercator; ExploraNova, La Rochelle, France). In the Fos/GAD and the Fos/MCH studies, Fos single- and double-labeled neurons were plotted on sections taken at 600 µm intervals (6 sections between AP −2.00 and −5.00 from Bregma) for 4 rats per condition. For the MCH/GAD study, MCH and MCH/GAD neurons were plotted on sections taken every 300 µm (7 sections/rat, between AP −2.10 and −3.90 from Bregma). In all cases, the number of plotted neurons per structure was counted using Mercator (ExploraNova). When a structure was present on several sections the neurons counted on all sections were summed. The atlas of Paxinos & Watson [51] was used as a reference.

### Statistical analysis

Since we compared distribution-free small-size samples, we performed non-parametric tests and reported median and quartiles data. Kruskal-Wallis tests were performed on vigilance state quantities and number of labeled neurons for each structure across experimental conditions (PSC n = 4, PSD n = 4 and PSR n = 8). Post-hoc Mann-Whitney tests were used to identify significant pairwise differences (PSR or PSD vs PSC; PSR vs PSD). All statistics were performed using StatView v.5.
